# Social position of older immigrants in the Netherlands: where do immigrants perceive themselves on the societal ladder?

**DOI:** 10.1007/s10823-022-09453-3

**Published:** 2022-04-20

**Authors:** Silvia S. Klokgieters, Theo G. van Tilburg, Dorly J. H. Deeg, Martijn Huisman

**Affiliations:** 1 Department of Sociology, Faculty of Social Sciences, Vrije Universiteit, De Boelelaan 1081, 1081 HV, Amsterdam, The Netherlands; 2 Department of Epidemiology & Data Science, Amsterdam Public Health Research Institute, Amsterdam UMC - location VUmc, De Boelelaan 1089a, 1081 HV , Amsterdam, The Netherlands

**Keywords:** Subjective social position, Older immigrants, Socioeconomic circumstances, Life course

## Abstract

Older Turkish and Moroccan immigrants are often ascribed a low social position based on their relatively unfavourable educational level, occupational status and income. Yet immigrants emigrated to improve their social position and came from contexts where determinants of social position might be based on different socio-cultural circumstances than those used in the country of settlement. In order to understand immigrants’ own perception of their social position, we interviewed 23 60–68 year old immigrants from Turkish and Moroccan origin in the Netherlands. Using a ten rung ladder, participants were asked to position themselves in the societal hierarchy before migration, after settlement and currently. Most participants positioned themselves at a middle or high position on the societal ladder. Circumstances used for positioning were related to socioeconomic indicators, but also to social affirmation, family, social integration, physical, mental health, happiness and complying to religious prescriptions. When these circumstances were deemed favourable, participants tended to position themselves higher. Our findings also show that the circumstances that participants used for positioning themselves varied across the life course. These findings complement the picture of the often low objective low socioeconomic position of older immigrants and show that immigrants’ perception of their subjective social position reflects a broader set of circumstances than just socioeconomic ones.

## Introduction

Like many Western European countries, the Netherlands is faced with both the ageing of the population and an increasing ethnic diversity of the older population (Zubair & Norris, 2015; Ciobanu et al., 2016; Schellingerhout, 2004). Especially former labour immigrants are making up an increased share of the urban older population (Fokkema & Conkova, 2018; Schellingerhout, 2004). These immigrants predominantly came from Turkey and Morocco and have moved to the Netherlands for reasons of performing physical labour in the 1960 and 1970 s and family reunification in the 1980s. Former labour immigrants occupy a relatively low socioeconomic position in the country of settlement (Snel et al., 2007). They often migrated from poorer socioeconomic contexts and received little to no schooling. Upon arrival in the country of settlement, they have often performed hard physical labour for a large portion of their life (Guiraudon, 2014). As a consequence, many report a poorer health than their native peers, which makes them a relatively vulnerable group in old age (Hoogendijk et al., 2021; Klokgieters et al., 2018)

While many studies have focused on immigrants’ objective components of social position, reflected by indicators of education (Solé-Auró & Crimmins, 2008), occupation (Ballarino & Panichella, 2015; Snel et al., 2007), and income (Assari, 2020), there is a paucity of research that explicitly focuses on immigrants’ subjective social position. Yet, subjective social position is an important outcome in its own right and has been viewed as an important predictor of health (Singh-Manoux et al., 2003), psychological functioning (Adler et al., 2000) and labour market outcomes (Lindemann, 2007). In addition, a low subjective social position may lead to feelings of frustration or relative deprivation. When subgroups within societies consistently ascribe themselves lower positions, this may be a cause of social unrest, polarisation and lacking social cohesion (Lindemann & Saar, 2014).

There are three other reasons why studying subjective social position among older immigrants is important. First, current studies focusing on subjective social position have been predominantly quantitative (Engzell & Ichou, 2020; Lindemann & Saar, 2014; Singh-Manoux et al., 2003). While such studies have yielded important findings, such as establishing the link between subjective social position and health (Singh-Manoux et al., 2003), it is unclear how individuals perceive, understand and interpret their own social position. Particularly, the circumstances used by people to infer their own social position may be different from those predominantly used by researchers, who often refer to the traditional socioeconomic characteristics of educational, occupational and income level. For example, it has been argued that factors such as religion, gender and ethnicity play a key role in people’s perception of their own social position (Goldman et al., 2006; Nielsen et al., 2013).

Second, current studies that focus on subjective social position are predominantly conducted among non-immigrant majority populations (Lindemann & Saar, 2014). Therefore, little is known about how migration experience affects the subjective social position of immigrants (Engzell & Ichou, 2020). It has been argued that immigrants’ perception of their own social position may be ambiguous because they simultaneously position themselves on a lower rung in the country of settlement and on a higher rung in the country of origin (Fresnoza-Flot & Shinozaki, 2017; Ostrove et al., 2000). To date it is unclear how this ambiguity is perceived by immigrants themselves and how it affects their judgement of their own position in society.

Third, current literature on subjective social position focuses predominantly on younger and middle aged populations (Singh-Manoux et al., 2003; Wolff et al., 2010). This primary focus on younger adults precludes an understanding of the experience of people’s social position as it progresses across the life course. For instance, Wolff et al., (2010) suggested that temporal comparisons (current and past performances) play a unique role in the perception of social position. Thus, perceptions of one’s own social position may vary across the life course depending on the stage of life one is currently in. How social position is perceived among older adults, whose perspective may involve comparisons across the life course, has received scant attention in the literature so far.

The present study draws attention to the varied and dynamic nature in which the position on the social ladder may be perceived among older individuals with a migration background. Particularly, we focus on three questions (1) Where do older immigrants position themselves on the societal ladder? (2) Do they have traditional socioeconomic circumstances in mind when positioning themselves, or are other considerations important to them? If so, which ones? (3) Does their perception of social position change when it refers to different stages in their life course (i.e. currently, right after migration and before migration)? In what follows, we introduce the concept of subjective social position and elaborate on theoretical arguments about how circumstances, the migration experience and the life course may affect immigrants’ subjective social position.

### Defining social position

Studies of social stratification have used objective and perceived approaches to describe an individual’s position in society. Objective approaches often refer to educational, occupational and income level or other aspects related to one’s occupation (Singh-Manoux et al., 2003). Critiques on this objective measurement of social position are that it neglects how individuals themselves experience their rank in society and how individuals weigh aspects of status and relative deprivation in their experience (Lindemann & Saar, 2014).

Subjective social position is defined as “a person’s belief about his location in a status order” (Davis, 1956, p. 154) and is often measured by an instrument capable of conveying a hierarchal position in a larger social structure, for instance a ladder (Adler et al., 2000) or a pyramid (Evans & Kelley, 2004). Such a positioning involves social comparison as a fundamental psychological mechanism that links an objective description a subjective evaluations (Lindemann & Saar, 2014). Social comparison (i.e. comparisons of self to others) may involve various components including appraisals (i.e. how one perceives that others see oneself) and adaptive expectations (i.e. based on one’s own past performance current and future positioning is determined) (Franzini & Fernandez-Esquer, 2006).

### Subjective social position among older immigrants

Immigration background and ageing are likely to influence processes of social comparison in determining one’s own position and how one perceives that others sees her/him/them in the societal hierarchy in three ways. First, Singh-Manoux et al., (2003) have shown that self-assigned social position involves cognitive averaging of traditional indicators of socioeconomic position (i.e. education, occupation and income). It is unclear how important traditional socioeconomic indicators are in immigrants’ evaluation of their social position. In agricultural societies, such as Turkey and Morocco, where many labour immigrants in the Netherlands originated from, markers of status might be readily associated with other indicators like land ownership or property ownership (Kaya, 2008). Moreover, societies in Turkey and Morocco place particular emphasis on gendered status obtainment through marriage and Islamic religiosity that pervade cultural practices (Buitelaar, 2006; Uğurlu, Türkoğlu & Kuzlak, 2018). In these conditions, males might be awarded a higher status on the basis of being a father, breadwinner and householder whereas females are awarded a higher status when they are a mother, self-sacrificing and nurturing (Uğurlu, Türkoğlu & Kuzlak, 2018). Another way in which status might be awarded is through age. According to Yerden (2013), less industrialized countries tend to award a higher status to older people on the basis of their wisdom, respect and calmness. All these aspects may come into play when immigrants are asked to determine their own social position, especially immigrants approaching old age.

The second point is that people’s perception of their social position may change throughout the life course (Singh-Manoux et al., 2003). Subjective social position involves aspects of personal growth, future prospects or positive past experiences (Woo et al., 2017). With regard to immigrants, it is likely that both age and migration simultaneously influence both where and how they perceive themselves in the societal hierarchy. Individuals who seek to migrate may do so because they have hopes for a better future for themselves and their children (Woo et al., 2017). In this context, it may be critically important to have a job that serves the purpose of fulfilling these hopes. Older age, by contrast, may be a moment to evaluate and critically reflect on these choices. Particularly, as immigrants age, they cease to be labourers. This may lead them to re-evaluate their status, with doubts whether to stay or leave the country of settlement (Bolzman et al., 2006). Moreover, these considerations may cause them to evaluate the achievements of their children in conjunction with their own in order to justify their decision to migrate, or to reflect on their subjective social position (King et al., 2017).

Third, the referent group with which immigrants compare themselves might be different because of migration. According to social comparison theories, people tend to compare themselves to similar others in order to find their position within a social order (Wolff et al. & Kawachi, 2010). Immigrants may compare themselves to other immigrants living in the country of settlement (Leach & Smith, 2006), or they may compare themselves with individuals who stayed behind in the country of origin (Fresnoza-Flot & Shinozaki, 2017). The concept of varying referent groups among immigrants have been observed for instance through a ‘transnational expenditure cascade’ for Vietnamese women living in the U.S. (Thai, 2014) and female domestic workers from Filipino decent (Parreñas, 2001). The concept of a cascade refers to the idea that immigrants in the aforementioned studies improved their subjective social position in the country of origin substantially after migration while still occupying a disadvantaged position in the country of settlement. The choice of the comparison group has consequences for the position that immigrants ascribe to themselves on the societal ladder.

## Methods

### Data Collection

Perceptions of social position are investigated among older Turkish and Moroccan immigrants living in the Netherlands. Turkish and Moroccan immigrant participants were selected from a larger sample originating from the Longitudinal Aging Study Amsterdam (LASA) (Hoogendijk et al., 2019). Starting in the 1960’s, predominantly Turkish and Moroccan men came to the Netherlands to perform (mostly) physical labour. As of the 1980s, many wives and children from Turkey and Morocco followed their husbands. To date Turkish and Moroccan immigrants are often not represented in study samples among the general older population due their small numbers, language barriers and experiences of stigma and marginalization. Therefore, these groups were purposively sampled for LASA. This sample included a total of 478 immigrants. From the original sample eighty participants were approached to partake in the current qualitative study. Twenty were not included because they were not at home at the time of the visits nor reachable over the phone. Thirty-seven refused to participate in the interview for various other response reasons. For example, being physically incapable or having already received many requests for interviews. Participants who were selected were sent a letter for notification and were visited a week later together by the first author and research-assistants with Moroccan Arabic or a Turkish-speaking ability. In total, 23 participants were interviewed, ten of which were born in Turkey and thirteen in Morocco (Table 1).

**Table 1:** Characteristics of participants

Pseudonym	Employed (No/Yes)	Educational level (N/L/M/H)	Income (€ net per month)	Country of birth (T/M)	Reason migration	Married (No/Yes)
Mr A	No	L	795–901	T	Work	No
Mr B	No	M	2043–2269	T	Other	Yes
Mr C	No	L	1135–1361	M	Family	No
Mr D	No	N	1816–2042	T	Work	No
Mr E	No	N	1135–1361	M	Work	No
Mr F	No	L	795–907	M	Other	No
Mr G	No	L	3177–3403	T	Work	Yes
Mr H	No	L	568–680	M	Family	No
Mr I	No	L	2043–2269	M	Work	Yes
Mr J	No	L	1589–1815	T	Work	Yes
Mr K	No	L	1362–1588	T	Work	No
Mr L	No	L	1135–1361	M	Family	Yes
Mrs M	No	N	795–907	M	Family	No
Mrs N	No	M	3631–3857	M	Family	Yes
Mrs O*	No	N	795–907	M	Family	No
Mrs P	No	H	Refusal	M	Family	No
Mrs Q*	No	L	Does not know	M	Family	No
Mrs R	No	N	1362–1588	T	Family	Yes
Mrs S	Yes	M	1589–1815	M	Family	Yes
Mrs T	Yes	L	1135–1361	M	Family	No
Mrs U	No	N	1135–1361	T	Family	Yes
Mrs V	No	L	1816–2042	T	Other	Yes
Mrs W	No	L	1022–1134	T	Other	Yes

Notes. N = no education, L = low, M = middle, H = high, T = Turkey, M = Morocco

### Role of research-assistants

In order to interview Turkish and Moroccan immigrants in their own language we collaborated with two bilingual research-assistants. They were elaborately briefed on the study topic and aims. During the translation of the topic list, word usage was discussed in all three languages (Dutch, Turkish and Darija) in order to reach agreement across interviews. During the interview, they asked clarifying probing questions if necessary and to give a summary of the participant’s answer. Summaries were only preferred when the participant’s answer was very long or when the participant was emotional. In other cases the participant’s whole answer was translated. We worked collectively during the interview. The principal researcher had a coaching role in initiating and determining the new topics, supervising the assistants, and asking probing questions.

### Interviews

**Fig. 1:**
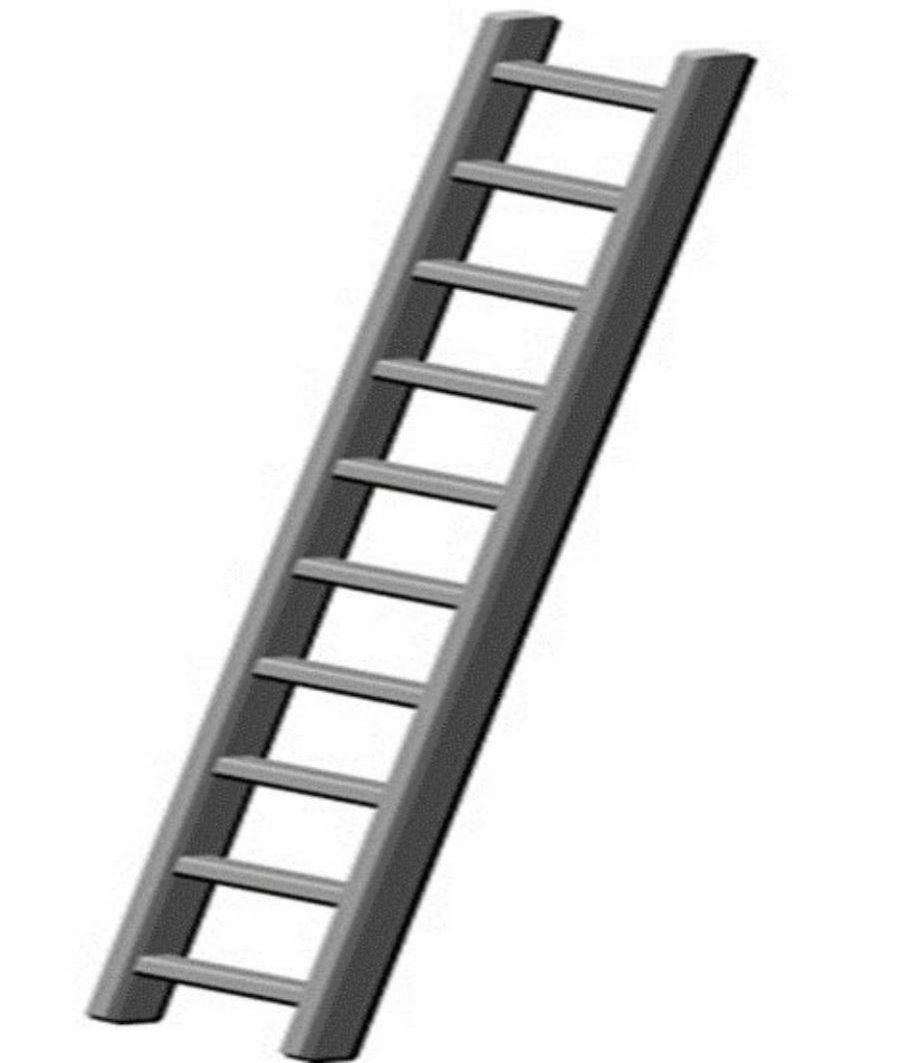
The ‘Societal Ladder’

We applied an increasingly rigid interviewing structure. Interviews were initiated by asking a broad open question “What do you feel is going well in your life currently?” Subsequently, a number of tools were used. For this study we focus on the results obtained through the ‘community ladder’ (Adler et al., 2000), here referred to as the societal ladder. The participant was shown a picture of a ladder with ten rungs (Fig. 1; Adler et al., 2000) with an explanation that those who were best off in society were at the top of the ladder, and those who were worst off were at the bottom. We made no reference to ‘traditional’ socioeconomic or to other factors. The participant was, subsequently, asked to position him/herself on this ladder reflecting their current situation, their situation before migrating and their situation right after migrating, and was asked why he or she chose the positions.

The interviews lasted between one and two hours. Apart from the interviews of two participants who refused, all interviews were tape-recorded. The tapes were transcribed and translated verbatim. Translations from Turkish or Daija to Dutch were executed by research-assistants. The other interviews were summarized based on notes taken during the interview. As recommended by Van Nes et al., (2010), for the translations of quotations from Dutch to English the first author worked side by side with a native English editor skilled in Dutch.

### Analysis

We used thematic analysis which serves the purpose of identifying, analysing and reporting patterns (themes) (Braun & Clarke, 2006). Firstly, transcripts were (re)read several times in order to derive initial codes. We focused on instances where the participants made referrals to their social position in the initial part of the interview and in the part where the ‘societal ladder’ was used. We payed specific attention to positioning strategies, which mentioned specific circumstances or referent groups and whether positioning strategies resulted in ascending, descending or stable positions on the ladder. Next, the initial codes were analysed for the purpose of understanding the relationship between codes and to distil themes and levels of themes. We used methods of constant comparison by rereading and recoding the interviews to ensure consistency in coding and analysis. Finally themes of social position lead to an insight into how immigrants positioned themselves at various positions across the life course.

## Results

Participant characteristics are provided in Table 1. Most participants had a low level or no education. The average net monthly income was in the category from €1194 to €1637. Most participants were retired and all had one or more children. Most men migrated for reasons of work and most women migrated because of family reunification.

### ‘Participants’ Current Perception of their Social Position: Low or High?

In the first research question we asked where participants positioned themselves on the societal ladder. Table 2 depicts the rung where participants currently positioned themselves. Among male participants, there was a large variation: participants positioned themselves across the full range of the ladder. All females positioned themselves higher than the fifth rung of the ladder. Combinations of relatively favourable objective socioeconomic indicators and a low subjective position on the societal ladder, and vice versa, were observed.

**Table 2:** Participants placement on the societal ladder

Pseudonym	Currently	Right after migration	Before migration	Socioeconomic	Social	Societal	Health & Wellbeing	Religion
Mr A	1	4	10	X	X			
Mr B	1	5	10		X		X	X
Mr C	1	9	10	X				
Mr D	Varies†	1	5		X	X		
Mr E	5	5	5	X				
Mr F	10	10	10	X	X		X	X
Mr G	10	5	1	X		X		
Mr H	10	Varies††	10	X		X		
Mr I	5	3	1	X				
Mr J	5	5	4	X		X	X	
Mr K	5	4	1	X	X		X	
Mr L	10	5	1	X				X
Mrs M	5	10	6		X			X
Mrs N	8	4	7	X		X		X
Mrs O*	5	1	10		X	X	X	
Mrs P	9	5	5		X			X
Mrs Q*	10	10	10					X
Mrs R	10	10	10	X	X			X
Mrs S	7	6	6	X	X		X	
Mrs T	7	4	2			X	X	X
Mrs U	10	10	1		X		X	X
Mrs V	10	5	5	X	X			
Mrs W	7	6	*		X		X	

Notes. * Participant did not answer the question. † Participant based his position on the weather and whether he feels healthy: when the weather is good and he is healthy he places himself high, when conditions are normal (according to him weather and health not extremely bad) he places himself in the middle. †† Participant based his position on the country of residence after migration. In Spain: first high and then low; in France: low; in Belgium: high and then later low; in the Netherlands: high

### Socio-Economic Circumstances or other considerations

The second research question asked whether participants, currently, mainly had in mind socioeconomic circumstances or whether they additionally or exclusively used other circumstances to position themselves. Note that socioeconomic circumstances were not mentioned during the interview. As such, the circumstances that are mentioned here are based on participants’ own considerations. Table 3 includes the themes that were mentioned during the interview, which can be summarized as socioeconomic, social, societal, health & wellbeing and religious domains.

**Table 3:** Explanation of circumstances

Theme	Codes
Socioeconomic	Employment, being smart (as a substitute for being educated), being rich, having enough money to live from, status, property ownership, coming out of poverty
Social	Being married, having a good marriage, having (married) children, having (grand)children, receiving affirmation by others, good interaction with others
Societal	Freedom, opinion of society, language, knowledge about culture, ability to participate in society, homesickness, feeling fearful and alone, learning a new culture
Health and wellbeing	Being healthy, feeling healthy, being happy, feeling young, being a good person, being sad
Religion	Going to Mecca, praying regularly, feeling close to God, position to God, knowledge about the religion, being a good Muslim

Socioeconomic circumstances were often used for self-positioning, and therefore played an important role, participants mentioned them in conjunction with other circumstances (see Table 3).

Regarding the socioeconomic domain, Table 3 depicts which socioeconomic circumstances were mentioned. Participants referred to multiple socioeconomic circumstances including, employment, education, being rich, and having enough money to live from. Mr E, for example, focused primarily on employment:

*‘I already said in the middle, now too. Sometimes in Morocco, sometimes in the Netherlands. If you don’t work, you go down.’* (Mr E, currently on rung 5).

Mrs N mentioned that she would have achieved a higher rung in her life, if only she had studied more. Consequently she positioned herself not on the tenth but eighth rung:

*‘Sometimes I think “oh if I would’ve studied”, a little bit language, then I would’ve had a better job than the one that I have now. I’d be a doctor or something else, a psychologist’* (Mrs N, currently on rung 8).

More, often, however, participants focused on being smart rather than their education as a means of positioning. Mr I, for example, positioned himself on the fifth rung because he feels he is less smart but not in a particularly bad position economically:

*‘Yes, perhaps this other person has studied a lot and then you have, “This is your salary”, done. And yes, the other person didn’t study. He’s just a smart cookie. You know, he’s even better off than the person who studied and he has enough money to live on [.] Look, when you’re there [rung 10], then you have to think a lot. I find it difficult, thinking; I’m not that kind of person.*’ (Mr I, currently on rung 5).

Mr C, by contrast, focused on wealth. He explained that those who are rich are on the highest rungs of the ladder and those who are poor on the lowest rung. He positioned himself on the lowest rung because he considered himself to be poor.

*‘Financially I cannot do that, and yes, all problems in life start from that point, financially. If I stay here [rung 1]. [.] It’s not so much, but I’ve learned to live on it.’* (Mr C, currently on rung 1).

While Mr C assigned himself to the lowest rung of the ladder, he still emphasized that he learned to live with his situation and was able to sustain himself with little money. This was narrated by many participants, who admittedly were not so well off socioeconomically, but who emphasized being content with their situation. Women also sometimes mentioned the importance of wealth. Mrs R, for example, mentioned she was happy that her husband gave her enough money and she had freedom in spending it. Therefore, she positioned herself on the tenth rung of the ladder:

*‘My husband never asked me what I did with the money. Where I spent it or how much of it was left. I sometimes hear from people around me that this happens.’* (Mrs R, currently on rung 10).

Within the social domain various circumstances were mentioned, including being married, having a family life (children and grandchildren, preferably married) and being appreciated by others through social affirmation (Table 2). In this regard participants seemed to have a vision of what their situation ought to be. For example, they wanted a wife, children and grandchildren. Participants positioned themselves on the ladder according to the extent to which they saw themselves fulfilling this vision. Mr K and Mr B provided examples for this line of reasoning.

Participant: *‘The middle, in the middle.’*

Interviewer: *‘And why there?’*

Participant: *‘Because I don’t have a wife. If I had a wife, then I would be higher.’* (Mr K, currently on rung 5).

*‘Myself? I am all the way down here [rung 1]. This is my life. Look no wife, wife died, no money, nothing. […] A marriage is better, your wife makes tea in the morning, prepares breakfast.’* (Mr A, currently on rung 1).

Mr K considered his socioeconomic circumstances to be not too bad. As a consequence, he positioned himself close to the middle of the ladder. What refrained him from positioning himself higher is that he did not have a wife. Mr B also referred to money, but singled out his lack of marriage as a reason for his low position. This represented a broader vision in which marriage is an important precondition for a higher position in the societal hierarchy.

Another way in which social circumstances played a role in self-positioning was through social affirmation. Participants emphasized being good to others and feeling appreciated by others through statements such as “ask my uncle.” Mrs N, for example, positioned herself on the eighth rung of the ladder because of her educational level and wealth. In addition, she mentioned regularly how well others perceive her:

*‘All the way at the top. Why would I put myself at the bottom? I never treated anyone badly and I never put anyone down. [.] But also my neighbours, my colleagues, they say that they were happy with me.’* (Mrs N, currently on rung 8).

Within the health and wellbeing domain participants mentioned aspects of mental and physical health and happiness. In general, being unhappy, unhealthy and poor were associated with lower positions; being happy, healthy and rich were associated with higher positions. Some participants mentioned their health casually and in relation to their wealth. A participant said “*You have to be happy and rich*” (Mr J, currently on rung 5) and another said “*I am here, I am not sad and I am not sick*” (Mrs S, currently on rung 7). Other participants mentioned that health was actually the most important determinant of positioning, like Mr J.

*‘The best wealth is health. When I’m healthy, I’m rich. That’s the only type of wealth I see.’* (Mr J, currently on rung 5).

The religious domain included various aspects of religious practice that were often directly related to Islam, particularly in terms of being a “good Muslim”. Participants positioned themselves on the ladder based on whether or not they complied with pillars of Islam including pilgrimage to Mecca or praying five times a day. Combining the religion domain and the socioeconomic domain seemed to offer contradictory lines of reasoning. On the one hand, participants emphasized how unimportant money and status were to them: it was morally unjust to perceive these as important according to their religion. On the other hand, they used their socioeconomic circumstances as a means to position themselves. In order to reconcile religion with their position, they emphasized regularly how grateful they were with their life and how little influence they themselves had on achieving their socioeconomic position. Mr L, for example, used income and wealth as reasons to position himself on the ladder. Later in the interview he emphasized the role of religion in his wealth:

*‘Thank God, we have everything. May God make us rich in our faith. Our faith in God is strong.’* (Mr L, currently on rung 10).

Some participants felt that their religion impeded them in using socioeconomic circumstances for positioning. Instead, they offered alternative circumstances for self-positioning such as religion and happiness. One example is Mrs Q who rejected the importance of socioeconomic circumstances:

Interviewer: *‘Where do you see yourself on the ladder at the moment?’*

Participant: *‘Well, what I see, I’m a believer, so the societal ladder doesn’t mean anything to me. Absolutely nothing.’*

Interviewer: *‘Why not?’*

Participant: *‘You live, you have a roof over your head, like everybody else. To associate yourself with a ladder, doesn’t mean anything to me. I don’t know, if I look, for example, at the Prophet, he was a very poor man, he slept on the floor and sometimes he didn’t eat for three days.’*

But when she was asked to position herself on the ladder:

Participant: *‘Well, I* ***feel*** *I’m at the top.’*

Interviewer: *‘And what puts you there? What are the things …?’*

Participant: *‘Well, when you’re happy with yourself, then you’re the happiest person on earth, really.’* (Mrs Q, currently on rung 10).

It should be noted that the interviewer never mentioned socioeconomic circumstances. Yet, Mrs Q along with others felt compelled to explain why she herself did not want to use socioeconomic circumstances. Instead she resorted to her own view of social position which was related to happiness and being a good person.

### Changes over the life stages

The third research question was whether participants’ perception of their social position changed when referring to different stages in their life (i.e., currently, right after migration and before migration). We found that most of participants experienced a change in their subjective social position. In general we observed four patterns that included a declining subjective social position, an increasing position, a dip in position right after migration and a stable position (Table 4).

**Table 4:** Changes before, right after migration and currently

Theme	Characteristics
Pattern 1	Declining social position: Mr A, Mr B, Mr C,
Pattern 2	Increasing social position: Mr G, Mr K, Mr L, Mrs P, Mrs W, Mrs T, Mrs U, Mrs V, Mrs W
Pattern 3	Dip right after migration: Mrs N, Mrs O, Mr D, Mr J
Pattern 4	Stable social position: Mr E, Mr F, Mrs Q, Mrs R, Mrs S

Various participants experienced a declining subjective social position over different life stages. Participants justified changes using socioeconomic, social, age, and societal conditions. Mr C, for example, moved from rung 10 during his younger years and 9 right after migration, towards the lowest rung on the ladder during older age. He gave the following explanation:

Before I came to the Netherlands. I was here, at the top [..] I had two cars, two houses, my children went to the nicest kindergarten, there was enough money [..] this situation, where I am now, yes down, at rock bottom.

Mr C apparently felt he became much poorer after migration than before. As such, he positioned himself lower on the ladder in older age. Similarly, Mr A descends from the societal ladder from rung 10 to rung 4 to rung 1. In contrast to Mr C who focused on socioeconomic circumstances, Mr A focused on unrealized expectations in order to position himself:

In Turkey, I wanted to have a good life, of course. To have a family, to be happy – and then I could put myself right at the top. Now though, I have no house, no family, nothing. So I can’t be up there. I’m still all the way down there [points to the lowest rung of the ladder].

Another example in this category is Mr B who experienced disappointment, focused on the way he is treated by Dutch people, which he regards especially prevalent in later in life. He was bitter over a lack of acceptance and increased discrimination that he experienced in Dutch society. This is a reason for him to position himself lower on the ladder:

*‘I feel … second-rate, even a third-rate person. I used to feel like a Dutchman, nothing else. Okay, I was Turkish, but I had internalised it – I was Dutch. [.] My fear, my son has my last name, is that something will happen to him. Like what happened to the Jews… In this society, we are inferior…’* (Mr B, down from rung 5 to rung 1).

In the second pattern, participants moved up on the ladder in their perception. They used circumstances of socioeconomic, social, societal, health and well-being in order to explain their upward movements. Mr K experienced severe poverty during childhood:

*‘Well, back then, our financial situation was not so good. We couldn’t survive on what we had. We were a big family. The situation is good now’* (Mr K, up from rung 1 to rung 5).

While he had problems with providing food for his family currently, he had more severe problems before migration. He therefore moved from the first to the fifth rung. Mr K was not unique in this view of the ladder. Right after migration participants often mentioned that they saw themselves making upward movements because they now had a job and a stable income. This reflected a positive view on migration. Having work, regardless of the status of that work, was considered highly important to make slow ascending movements towards the top of the ladder.

*‘I slowly started working, make money. I worked about 13 or 14 years here. I sent money. So slowly I went up.’* (Mr L, up from rung 1 to rung 5).

Participants who perceived to be climbing up the ladder also used arguments related to religion as reasons for this perception to ascend the ladder. They positioned the experience of growing old in the perspective of their religion and felt that they came closer to God as they aged. Knowledge attainment was important in this regard and granted them the reasons to move to a higher rung on the ladder.

Interpreter: *‘You said you were “still fighting.” What do you mean by that?’*

Participant: *‘In terms of knowledge. Knowledge about religion. [.] My lifestyle changed to a lifestyle that better fits my religion.’* (Mrs W, up from rung 6 to rung 7).

Societal conditions also were referred to in moving upward on the ladder. For some female participants, the sense of freedom was important for moving upwards. Mrs P, for example, refused to see herself in a good position during childhood because she felt that she was denied life experience:

*‘For us, in Morocco, for example, in my time, when you were, like, 19–20 years old, you weren’t “living.” You understand? Just to school, home. It is not like here, from 12 and 13 years old you can, for example, meet guys, go out in a group. With us, no.’* (Mrs P, up from rung 5 to rung 9).

She clearly refers to constraints imposed on her during her childhood in Morocco. These constraints hampered her to gain life experience and she gave herself a low social position as a consequence. Later in life, she mentioned that she moved up the ladder because she felt that she had gained freedom and life experience.

In the third pattern, participants experienced a brief ‘dip’ in their social position right after migration. After the dip, these participants perceived themselves to move back up on the ladder, often because they were habituated to their lives in the Netherlands. During the dip, participants mentioned fear, unfamiliarity with the Netherlands, bad weather and darkness as reasons to position themselves low. Participants also referred to a lack of access to services or social support, often resulting from a lack of language skills. An example is Mrs N who mentioned that she felt handicapped because of her limited language skills. During later life she positioned herself higher as she learned to speak Dutch and was independent (Mrs N: from rung 7 via 4 to 8). Other examples of participants with this pattern include Mrs O (from rung 10 via 1 to 5) and Mr D (5 via 1 to varied). Mr D, despite variations, emphasized the adjustments he needed to make after he migrated and how he perceived a recovery of status when he was more familiar with his current location. He mentioned:

*‘You don’t know anything, you don’t know how to buy bread, you can’t go anywhere. When you go to a café, you don’t say “coffee” but you say “khafe” and then they don’t understand you.*

(…)

*but we gotten used to the Netherlands now. For example, if you want to go to the doctor, you make an appointment and you go.’* (Mr D, from 5 to 1).

In the fourth pattern, participants described that their social position had remained unchanged in their lifetime. Rather than moving up or down, participants who remained at the same position used different circumstances each time they were asked to position themselves. In this way they were able to argue that they stayed in the same social position. An example is Mr F who clearly referred to socioeconomic circumstances in order to position himself before migration. These circumstances were favourable, which results in a high position. Right after migration he said that he had a nice group of friends and he was happy, which formed his argument to, again, position himself in the tenth rung. In older age, while he admitted that circumstances were not so favourable, he still positioned himself on the highest position because he accepted his fate. Similarly Mrs Q positioned herself invariably on rung 10 based on that she felt happy and she refused to rate her status against that of others for religious reasons.

## Discussion

We set out to answer the question of how Turkish and Moroccan older immigrants living in the Netherlands position themselves in the societal hierarchy. Participants often positioned themselves at middle or high position. For choosing a position, they used circumstances related to socioeconomic indicators but they also alternated between circumstances from other domains including social, societal, health and well-being, and religion. Moreover, in the participants’ view, their social position changed over successive life stages with some positioning themselves low before migration and high afterwards, others remaining in the same position, and others experiencing decline in their position over time. Our findings can be summarized into three key contributions to the literature.

First, despite socioeconomic circumstances playing an important role in the subjective social position of Turkish and Moroccan immigrants, we found other circumstances that were also important. In line with Nielsen et al., (2013) for instance, we found that cultural gender norms influenced immigrants’ interpretations of their social position. Several women in our study directly referred to their husband when discussing their access to income and the freedom in making expenses. This is consistent with results from research among female Asian immigrant women in the U.S. (Chen et al., 2009) and among women in the U.S., Australia and Norway (Baxter, 1994), showing that women are more likely than men to derive their socioeconomic status from their spouse’s status. In addition, we identified instances where individuals’ perceptions about an ideal family situations and their role within the family were used as criteria for positioning in the societal hierarchy. This is consistent with research that found that Turkish men may gain status based on fulfilling a role within the family, such as being a father and breadwinner, and women gain status if they are self-sacrificing and nurturing mothers (Cela & Fokkema, 2017; de Haas & van Rooij 2010; Uğurlu et al., 2018).

Similarly, in line Nielsen et al., (2013), we found that religion was mentioned as a consideration for choosing a position on the ladder. We found instances in which religion was used as a reason to reject the concept of social position altogether. This line of reasoning was based in the idea that comparing oneself to others is morally rejectable according to the principles of religion (Ahaddour, van den Branden & Broeckaert, 2018; Buitelaar, 2006). In these cases, individuals based their social position on being a good person (or Muslim) instead.

The second addition to the literature is that both migration and aging influenced immigrants’ perception of social position. The influence of migration was most pronounced within the societal domain. Particularly, we observed a pattern in which immigrants experienced a dip in subjective social position right after migration among our participants. They indicated unfamiliarity with the Netherlands and language barriers, which hampered them in participating fully in society. This aligns with acculturation theories about migration in which immigrants initially experience a “culture shock” (Berry et al., 1987a; Bhugra & Becker, 2005). The influence of aging can best be captured in the theme of health and wellbeing. Good overall health, i.e., physical capacities, but also happiness and mental health, was often mentioned in the context of a high position and, vice versa, poor health in the context of a low position. Older adults tend to be confronted with physical deterioration as they age (Stuck et al., 1999), which is known to be particularly prevalent among older immigrants (Klokgieters et al., 2018). Health and well-being are known to play a role in older adult’s general evaluation of aging (Bowling, 2008) and the results of our study show that they influence immigrants’ assessment of their subjective social position as well.

The third addition to the literature is that we have not observed any instances where immigrants used a specific reference group for positioning oneself. This contrasts with many studies (Leach & Smith, 2006; Wolff et al., 2010) showing the importance of the referent group for self-positioning. Instead, immigrants predominantly looked to their own experiences and were able to compare their own circumstances over time. This confirms the idea that subjective social position involves aspects of personal growth, future prospects or positive past experiences (Woo et al., 2017).

One important limitation of our study was that in some cases the ladder instrument was hard to grasp for participants. Some initially understood the ladder as referring to the timing of migration or had trouble with the ladder as representation of their social position. This raises questions about the applicability of the ladder instrument, particularly given that some immigrants in this study were low-skilled and illiterate (Glasner et al., 2015). However, we believe that all participants had a sufficient understanding of the ladder to draw conclusions about their social position, although it took several tries and explanations by the interviewer in some cases. In addition, alternative understandings also provided novel insights in the way in which our participants viewed their own social position. For example, the participants who actively rejected socioeconomic circumstances as a means of positioning themselves revealed that they had knowledge about what the common denominators of social position are in the country of settlement.

Based on the findings of this study we draw two overall conclusions about immigrants’ subjective social position. First, immigrants’ subjective social position is dynamic over the life course. It takes into account aspects of migration and sociocultural integration, which are specific to each immigrants’ personal migration history. Second, immigrants’ own view of their social position is comprehensive and reflects circumstances that are situated in multiple life domains, i.e., socioeconomic as well as social, health and well-being, and religious domains. We have identified dimensions of both marginalization and privilege that play a role in where immigrants position themselves in the social hierarchy. Many immigrants perceived their social position as favourable despite seemingly unfavourable socioeconomic circumstances.
